# Urban Development in Sub-Saharan Africa: Bearer of Goods and Risks

**DOI:** 10.1371/journal.pmed.1001684

**Published:** 2014-07-29

**Authors:** Fahad Razak, Lisa Berkman

**Affiliations:** 1 Harvard Center for Population and Development Studies, Harvard University, Boston, Massachusetts, United States of America; 2 Li Ka Shing Knowledge Institute, St Michael's Hospital, University of Toronto, Toronto, Canada

## Abstract

Fahad Razak and Lisa Berkman discuss the implications of the study by Riha and colleagues for research into urbanization and the development of noncommunicable diseases.

*Please see later in the article for the Editors' Summary*

**Linked Research Article**

This Perspective discusses the following new study published in *PLOS Medicine*:

Riha J, Karabarinde A, Ssenyomo G, Allender S, Asiki G, et al. (2014) Urbanicity and Lifestyle Risk Factors for Cardiometabolic Diseases in Rural Uganda: A Cross-Sectional Study. PLoS Med 11: e 1001683. doi:10.1371/journal.pmed.1001683

Johanna Riha and colleagues evaluate the association of lifestyle risk factors with elements of urbanicity, such as having a public telephone, a primary school, or a hospital, among individuals living in rural settings in Uganda.

Sub-Saharan Africa remains the least urbanized region of the world and more than 60% of the population, 570 million people, still live in rural areas [1]. Over the next few decades Africa will be one of the most rapidly urbanizing regions [2], and with this transition is an expected rise in cardiovascular risk factors and disease (CVD) [3]. Across sub-Saharan Africa, many adults migrate back and forth from rural home communities to more urban areas for work and education; others have moved to urban areas; and in still other cases, rural communities themselves have urbanized. In this issue of *PLOS Medicine*, a study by Riha and colleagues is directly concerned with the latter scenario within the context of urbanizing rural Uganda [4]. As the authors aptly note, the crude dichotomy of urban-rural difference obscures the changes occurring within rural regions themselves, as characteristics of urban environments, defined as urbanicity [5], become more prominent. Urbanization is a complex worldwide phenomenon and challenges global populations to re-calibrate a set of far reaching behaviors as the meaning of communities change, networks widen, and globalization influences attitudes and access to new resources. Some of these phenomena are likely to be health promoting, while others expose formerly rural populations to new risks.

Riha and colleagues' study is the first in Sub-Saharan Africa, to our knowledge, to examine how urbanicity relates to the development of CVD risk factors in rural regions [4]. It is an important and revealing study. The population was drawn from 25 Ugandan villages that were unambiguously rural by conventional standards. A previously developed multi-country urbanicity scale was applied to score each village on seven domains meant to capture the hallmarks of urbanization: increasing population size and density, declining role of agriculture as the principal source of employment, rising education and diversity in educational achievement, increasing access to electricity and modern sanitation, and the presence of communication infrastructure [5],[6].

Compared to villages in the lowest quartile of urbanicity (most rural), individuals living in villages in the highest quartile (least rural) had a 50% increase in overweight, more than a 3-fold increase in heavy drinking, and were about 20% more likely to have low physical activity levels or a diet low in fruits in vegetables. This association showed minimal attenuation despite adjustment for individual level socioeconomic status (SES) quantified through a household asset and wealth index. There was no difference in smoking prevalence or hypertension. Given the great variability among countries in sub-Saharan Africa, it is unclear how generalizable these results are beyond Uganda.

## Urban-Rural Dichotomies: Too Simplistic to be Meaningful?

These results suggest a much more complex story than what is typically captured through well-trodden urban-rural classifications. The current dominant urban-rural dichotomy can be traced to at least the 1940s when the United Nations began reporting population statistics using this classification scheme [7], and is perhaps a legacy of a time when differences between urban and rural areas were much more discrete. However, the current application of these definitions varies widely. In a review of United Nations data on 228 countries about 50% used a strictly administrative criterion for urban (e.g., the capital city is by definition urban), 51 countries used a combination of size and density characteristics, 39 used type of economic activity, 22 had no definition of urban, some defined the country as completely urban (e.g., Singapore) and some as completely rural (e.g., some Polynesian countries) [5].

The between-country variability in definitions reflects the dynamic and context-dependent nature of what constitutes an urban environment, but it also implies that a blind application of these definitions to look at patterning of health outcomes is too simplistic. Multi-component urbanicity scales move beyond this dichotomy and allow us to understand what elements of an urban environment correlate with health outcomes, and perhaps gain a causal understanding of how this occurs. The principle advantages of these scales over basic urban-rural classifications are numerous: a much more precise and nuanced classification of communities on a full range of characteristics, detection of non-linear effects on health, detecting changes within communities over time in elements of urbanicity, increased statistical power to detect the impact of urbanicity on health, and potentially greater comparability of effects both within and between countries [6],[7].

## Disentangling the Impact of Socioeconomic Status on Health in Urbanizing Environments

Some hallmarks of rising urbanicity in low- and middle-income countries (LMICs) are increasing educational attainment and wealth, and transition in primary occupation away from agriculture. These aggregate village level metrics have entered the scoring system of urbanicity scales, including the scale applied in the paper by Riha and colleagues [6],[8]. Occupation, educational attainment, income, and wealth are fundamental measures of SES, and how to model their effect on health while simultaneously considering them as markers of an urbanizing environment is conceptually complex, especially given individual measures of SES can largely explain urban-rural differences in health for some measures [9]. A further challenge is how to capture income and wealth in LMICs, often leading to use of an asset index as a proxy for wealth [9]. To disentangle these effects, Riha and colleagues kept aggregate education and occupation within the urbanicity scale, but removed measures of household wealth and quality, and instead used an asset-based measure of wealth as the individual level SES measure. Essentially, they considered aggregate education and occupation solely at the village level and wealth as the only individual level measure of SES. Given the complexity and interplay of these factors, an analysis modeling occupation, education, and wealth at both the individual and aggregate village level would allow examination of their relative impact. Such a multilevel approach would permit us to examine whether context adds to our understanding of health dynamics, net of individual characteristics.

## Positive and Negative Health Consequences of Urbanization

The study of urbanization as a risk factor for non-communicable disease follows the “urban health penalty” paradigm [10], the idea that urban environments convey risk and are associated with greater rates of many diseases. But urban environments can also be the source of much that benefits human wellbeing [11]. It is unlikely that access to health centers, schools, and improved sanitation themselves lead to poor health. Rather it seems most logical that unmeasured conditions associated with these indicators lead to increased cardiovascular risk. The authors have limited insight into these factors, which are perhaps related to changes in diet, easier opportunities to purchase alcohol, and less physically demanding occupations. What distinguishes features of urbanicity that have positive and negative impacts on wellbeing is far from clear. Although the stated objective of the study by Riha is to identify “potential avenues for intervention” in the urbanization process to help prevent CVD, there are few obvious targets for public health intervention.

Critically, the focus on emergence of CVD with urbanicity should not take away from the broader needs of the population being studied. For example, 80% to 85% of this rural population still have body weights in the normal to underweight range, and across all of rural Uganda prevalence of underweight exceeds overweight [9]. Even though a detectable gradient exists between elements of urbanicity and CVD risk factors, one should not interpret this as implying that population needs have now shifted towards CVD prevention. Urban development and increases in social resources related to education, disease prevention, and better opportunities for work hold important promises for LMICs still confronting the costs of poverty and lack of health protection infrastructure. A broader analysis of all sources of disease burden in this population is required to set disease prevention priorities [12].

## Abbreviations

CVD: cardiovascular disease
SES: socioeconomic status
LMICs: low- and middle-income countries

## References

[1] Sub-Saharan Africa (2014) World Development Indicators, The World Bank. Available: http://data.worldbank.org/region/sub-saharan-africa. Accessed 1 January 2014.

[2] United Nations Department of Economic and Social Affairs (2012) World Urbanization Prospects The 2011 Revision. New York: United Nations.

[3] Hernández AV, Pasupuleti V, Deshpande A, Bernabé-Ortiz A, Miranda JJ (2012) Effect of rural-to-urban within-country migration on cardiovascular risk factors in low- and middle-income countries: a systematic review. Heart 98: 185–194. 21917659 10.1136/heartjnl-2011-300599 PMC3272377

[4] Riha J, Karabarinde A, Ssenyomo G, Allender S, Asiki G, et al (2014) Urbanicity and lifestyle risk factors for cardiometabolic disease in rural Uganda: a cross-sectional study. PLoS Med 11: e 1001683. 10.1371/journal.pmed.1001683 PMC4114555 25072243

[5] Vlahov D, Galea S (2002) Urbanization, urbanicity, and health. J Urban Health 79: S1–S12. 12473694 10.1093/jurban/79.suppl_1.S1 PMC3456615

[6] Novak NL, Allender S, Scarborough P, West D (2012) The development and validation of an urbanicity scale in a multi-country study. BMC Public Health 12: 530. 22818019 10.1186/1471-2458-12-530 PMC3554435

[7] Dahly DL, Adair LS (2007) Quantifying the urban environment: a scale measure of urbanicity outperforms the urban-rural dichotomy. Soc Sci Med 64: 1407–1419. 17196724 10.1016/j.socscimed.2006.11.019 PMC2001275

[8] Jones-Smith JC, Popkin BM (2010) Understanding community context and adult health changes in China: development of an urbanicity scale. Soc Sci Med 71: 1436–1446. 20810197 10.1016/j.socscimed.2010.07.027 PMC2942954

[9] Neuman M, Kawachi I, Gortmaker S, Subramanian SV (2013) Urban-rural differences in BMI in low- and middle-income countries: the role of socioeconomic status. Am J Clin Nutr 97: 428–436. 23283503 10.3945/ajcn.112.045997 PMC3742298

[10] Andrulis DP (2002) The urban health penalty: new dimensions and directions in inner-city health care. acponlineorg Available: http://www.acponline.org/acp_policy/policies/urban_health_penalty_new_dimensions_directions_in_inner_city_health_care_1996.pdf. Accessed 1 January 2014.

[11] McMichael AJ (2000) The urban environment and health in a world of increasing globalization: issues for developing countries. Bull World Health Organ 78: 1117–1126. 11019460 PMC2560839

[12] Gwatkin DR (2013) Metrics matter: the case of assessing the importance of non-communicable diseases for the poor. Int J Epidemiol 42: 1211–1214. 24057997 10.1093/ije/dyt167

